# Relationship between brain volume reduction during the acute phase of sepsis and activities of daily living in elderly patients: A prospective cohort study

**DOI:** 10.1371/journal.pone.0284886

**Published:** 2023-05-16

**Authors:** Toru Hosokawa, Kosaku Kinoshita, Shingo Ihara, Katsuhiro Nakagawa, Umefumi Iguchi, Marina Hirabayashi, Tomokazu Mutoh, Nami Sawada, Tsukasa Kuwana, Junko Yamaguchi

**Affiliations:** Department of Acute Medicine, Division of Emergency and Critical Care Medicine, Nihon University School of Medicine, Tokyo, Japan; Seoul St. Mary’s Hospital, College of Medicine, The Catholic University of Korea, REPUBLIC OF KOREA

## Abstract

Brain damage in acute sepsis may be associated with poor long-term outcomes that impair reintegration into society. We aimed to clarify whether brain volume reduction occurs during the acute phase of sepsis in patients with acute brain damage. In this prospective, noninterventional observational study, brain volume reduction was evaluated by comparing head computed tomography findings at admission with those obtained during hospitalization. We examined the association between brain volume reduction and performance of the activities of daily living in 85 consecutive patients (mean age, 77 ± 12.7 years) with sepsis or septic shock. The bicaudate ratio increased in 38/58 (65.5%) patients, Evans index increased in 35/58 (60.3%) patients, and brain volume by volumetry decreased in 46/58 (79.3%) patients from the first to the second measurement, with significant increases in the bicaudate ratio (*P* < 0.0001) and Evans index (*P* = 0.0005) and a significant decrease in the brain volume by volumetry (*P* < 0.0001). The change rate for brain volume by volumetry was significantly correlated with the Katz index (ρ = −0.3790, *P* = 0.0094). In the acute phase of sepsis in this sample of older patients, 60–79% of patients showed decreased brain volumes. This was associated with a decreased capacity for performing activities of daily living.

## Introduction

Sepsis is the leading cause of admission to the intensive care unit (ICU) [1]. Although the number of patients with sepsis is steadily increasing [2], the number of patients who survive and are discharged from the ICU has also increased due to technological advances in intensive care [3]. However, the quality of life of these patients after ICU discharge is lower than that of healthy people of a similar age [4]. Long-term outcomes are poor, especially in severe cases, and the survival rate after septic shock is approximately 40% [5]. In particular, sepsis-associated encephalopathy associated with brain dysfunction has been regarded as an important pathological condition related not only to survival rates but also to reintegration of patients into society [6,7].

In recent years, early intervention in diagnosis and treatment has been promoted, aimed at improving sepsis outcomes, the survival rate of severe sepsis [3], and long-term outcomes such as cognitive impairment [8].

To this end, we hypothesized that brain damage, including brain volume reduction that occurs in the acute phase of sepsis, may be associated with poor long-term outcomes. Evaluation of ventricular enlargement is as important as the evaluation of memory impairment [9,10]; however, previous reports on organic brain damage, decreased brain volume, and ventricular enlargement after sepsis have only used computed tomography (CT) [11,12] or magnetic resonance imaging (MRI) [13,14] for evaluation at a single timepoint during inpatient treatment. Furthermore, there are no reports evaluating brain volume reduction or ventricular enlargement over time.

Therefore, this study aimed to clarify whether brain volume reduction occurs during the acute phase of sepsis by focusing on acute septic brain damage. Furthermore, we examined its clinical significance, especially the correlation between poor activities of daily living (ADL) function and brain volume reduction or ventricular enlargement in the acute phase of sepsis.

## Patients and methods

### Patients and protocol

This was a single-center prospective observational study conducted at the Nihon University Itabashi Hospital. This study was conducted in accordance with the tenets of the Declaration of Helsinki. Approval was obtained from the clinical research institutional review board of the Nihon University School of Medicine Itabashi Hospital (RK-170912-08), and written informed consent was obtained from each patient or their family prior to enrolment. Patients admitted to our ICU between March 2018 and March 2020 were included. All patients had a clinical diagnosis of sepsis and septic shock as defined by the 2016 international sepsis criteria (Sepsis-3) [15]. The exclusion criteria were as follows: age < 20 years, in-hospital onset of sepsis, transfer from another hospital and intervention before admission to our hospital, and a Katz score [16] ≤ 4 before admission, indicating poor ADL function. Patients with in-hospital onset of sepsis and those who underwent intervention before admission were excluded because their hemodynamics were stabilized with early fluid therapy during the acute phase of sepsis [15], making the evaluation of brain volume reduction difficult due to the increased brain water content after fluid therapy. Moreover, patients with a Katz score ≤4 before admission were excluded because poor ADL function before admission is generally related to that at discharge.

Vital signs at admission were recorded for all included patients. Blood tests were performed on admission (day 0) and on day 7, day 14, day 21, and day 28. These periodic tests were routinely performed to determine the course of sepsis treatment. We used these results to calculate the daily Sequential Organ Failure Assessment (SOFA) scores. Head CT was performed at the time of admission and at the time of symptom change or discharge, as appropriate for follow-up. Vital signs, CT images, and blood biochemical data were documented in medical records. The diagnosis of sepsis was based on vital signs and blood test findings on admission.

### Bacteriological assessment

Blood culture was performed at the time of admission for all patients. To confirm the diagnosis of infectious illness, blood and bacterial cultures of specimens taken from organs considered to be the focus of infection based on clinical findings, imaging findings, and laboratory data were assessed. Although all patients met the Sepsis-3 criteria and clinical criteria for infectious illness, a subgroup of patients with negative bacterial culture results were defined as cases of unknown origin. The group classification was based on whether the bacterial cultures of specimens taken from the sites considered to be of septic focus were positive.

### Assessment of brain volume reduction

We compared the head CT findings at the time of admission with those obtained during the course of treatment. Using these images, we investigated brain volume reduction, evaluated using the bicaudate ratio (BCR) [11,17], Evans index (EI) [18], and volumetry (Vo) [19,20]. A decrease in brain volume was defined as an increase in BCR and EI or decrease in the brain volume by Vo in the subsequent head CT compared with those in the initial head CT. BCR, EI, and Vo were defined as follows: BCR is the width between the anterior horns of the bilateral lateral ventricles divided by that of the cerebrum at the same height at the caudate nucleus level. It is used to evaluate frontal lobe volume loss; therefore, an increase in BCR implies a decrease in frontal lobe volume. EI is the maximum width between the anterior horns of the bilateral lateral ventricles divided by that of the cranial lumen at the same height. EI is often used to evaluate ventriculomegaly, but it is also used to evaluate brain atrophy [12]. An increase in EI is associated with a decrease in brain volume. Vo was calculated using an image processing workstation, Ziostation (Ziosoft, Inc., Tokyo, Japan) on CT images. All head CT images were transferred to Ziostation, where they were reconstructed [20]. Subsequently, whole-brain images were extracted and adjusted with a window value of 25–35 Hounsfield units, and the ventricular volume was subtracted to calculate the brain volume. All measurements were performed by two physicians, and the patients were blinded to the measurements.

### Assessment of ADL

ADL were assessed at discharge or patient transfer and were evaluated with the Katz index. Of the six functions in the Katz index, patients with moderate or severe disability (i.e., requiring assistance with at least two of the following: bathing, dressing, toilet use, transferring, continence, and eating) were excluded from the analysis [21,22].

To examine the association between brain volume reduction and ADL, the entire study population, including patients with brain volume reduction, was analyzed. We also compared demographic characteristics and ADL in the groups with and without brain volume reduction. Patients’ demographic characteristics were age, the Acute Physiology and Chronic Health Evaluation (APACHE) II score, the SOFA score, presence or absence of septic shock, length of ICU stay, length of intubation, presence or absence of disseminated intravascular coagulation (DIC), serum leukocyte and platelet count, and serum values for hemoglobin, hematocrit, albumin, total bilirubin, aspartate aminotransferase, alanine aminotransferase (ALT), sodium, potassium, urea nitrogen, creatine, lactate, bicarbonate, C-reactive protein, glucose, antithrombin 3, and uric acid. The scores and blood test values at admission were used for examination.

### Assessment of fluid balance

We investigated whether fluid balance affected the reduction in brain volume. The in-out fluid balance for 24 h after admission and on the day of the final CT was calculated and compared between groups with and without brain volume reduction.

### Statistical analysis

Statistical analysis was performed using JMP version 13 (SAS, Cary, North Carolina, USA). Collected measurements were analyzed for normal distribution using the Shapiro–Wilk test. A *P*-value <0.05 was considered statistically significant. Discrete variables are indicated by integer values (%). In the case of continuous variables, normally distributed data are shown as mean ± standard deviation and non-normally distributed data as median and interquartile range. The Wilcoxon signed-rank test was performed for the comparison of two groups of paired nonparametric data. Spearman’s rank correlation test was performed to evaluate correlations. The Mann–Whitney U test was performed for the comparison of the groups.

## Results

### Clinical features

**Fig 1:**
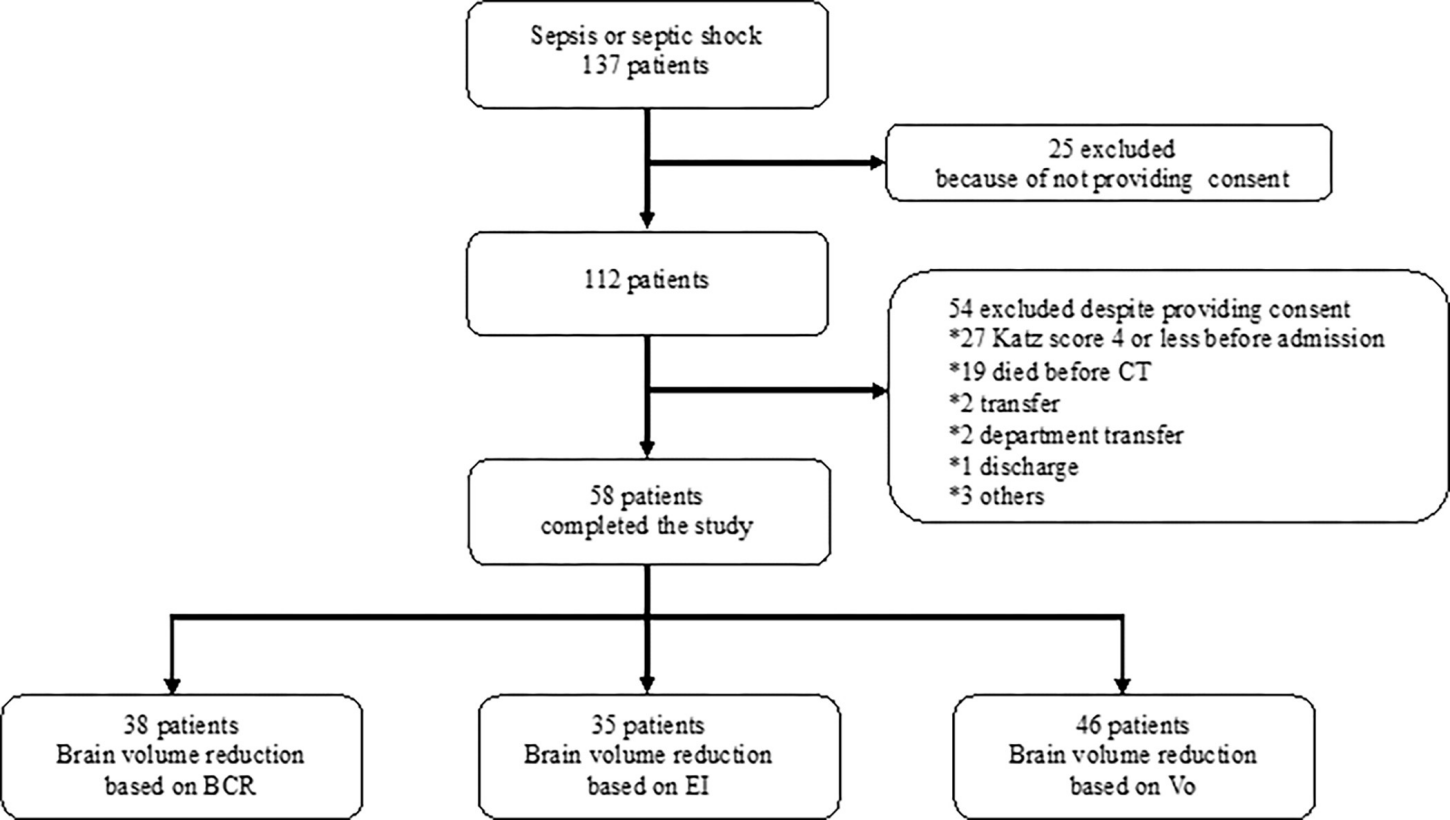
Flowchart of the inclusion and exclusion criteria. In total, 137 patients were diagnosed with sepsis or septic shock during the study period, of whom 112 patients provided informed consent for this study. Among these, patients with a Katz score ≤4 before admission, early death, hospital transfer, department transfer, and discharge were excluded; finally, 58 patients were included in this study; they underwent head CT at ≥2 timepoints, including the time of admission. In total, 38 patients had brain volume reduction based on the BCR, 35 patients had brain volume reduction based on the EI, and 46 patients had brain volume reduction based on Vo findings. Abbreviations: CT, computed tomography; BCR, bicaudate ratio; EI, Evans Index; Vo, volumetry.

Among the 137 patients with sepsis and septic shock admitted to our ICU between March 2018 and March 2020, consent was obtained from 112 patients. Patients whose Katz score was ≤4 before admission (n = 27) and whose brain CT could not be performed due to death (n = 19), hospital transfer (n = 2), department transfer (n = 2), discharge (n = 1), or other reasons (n = 3) were excluded. Thus, 58 patients were enrolled, and they underwent brain CT at two timepoints (Fig 1).

Clinical and demographic characteristics of the study population are presented in Table 1A. The mean age was 77 ± 12.7 years, 24 were women (41.4%), the APACHE II score at admission was 24 ± 7.0, SOFA score was 7 ± 3.2, and septic shock occurred in 17 (29.3%) patients. The length of ICU stay was 8 (5–12) days, length of hospital stay was 20 (12–32.25) days, and length of intubation was 2.5 (0–6) days. The mortality rate was 5%, tracheostomy was performed in 6 (10.3%) patients, and hemofiltration therapy for acute kidney injury was performed in 10 (17.2%) patients during hospitalization. DIC was observed in 25 (43.1%) patients during hospitalization. The mean ADL score at discharge or transfer was 1 (0–4) as per the Katz index. ADL function was evaluated at a median of 20 (12–32.25) days from admission. Original infections included pneumonia in 31 (53.4%) patients, urinary tract infection in 15 (25.9%), intra-abdominal infection in 6 (10.3%), soft tissue infection in 3 (5.2%), and others in 3 (5.2%) (Table 1B). The “other” category included one case each of periapical dental infection, infectious endocarditis, and bacteremia with unknown foci. Bacteremia was observed in 17 (29.3%) patients. The pathogens were pure gram negative in 20 (34.5%) patients, pure gram positive in 17 (29.3%), mixed bacterial in 13 (22.4%) patients, influenza virus in 2 (3.5%) patients, and *Candida albicans* in 1 (1.7%) patient. Pathogens could not be identified in 5 (8.6%) patients (Table 1B) because the specimens were insufficient for analysis in three cases and antimicrobial agents were administered before specimen collection in two cases.

**Table 1:** Characteristics and outcomes of the study population.

(A)	
**Variables**	**n = 58**
Age (years)	77 ± 12.7
Female, n (%)	24 (41.4)
APACHE Ⅱ score at admission	24 ± 7.0
SOFA score at admission	7 ± 3.2
Septic shock, n (%)	17 (29.3)
Days in the intensive care unit	8 (5–12)
Duration of hospitalization (days)	20 (12–32.25)
Duration of intubation (days)	2.5 (0–6)
Mortality, n (%)	5 (8.6)
Tracheotomy, n (%)	6 (10.3)
Hemofiltration, n (%)	10 (17.2)
DIC, n (%)	25 (43.1)
Katz score at discharge	1 (0–4)
Duration between the two CT scans (days)	13 (7–17)
WBC (×10^3^/μl)	10.75 (7.375–15.975)
Hemoglobin (g/dl)	13.15 (11.4–14.9)
Hematocrit (%)	40.55 (34.4–43.5)
Platelet (×10^4^/μl)	18.2 (12.75–27.9)
Albumin (g/dl)	3.3 (2.7–3.825)
T.Bilirubin (mg/dl)	0.65 (0.415–1.0075)
AST (U/l)	47 (24.75–85.5)
ALT (U/l)	28.5 (14–67.75)
Na (mEq/l)	138 (134.75–144)
K (mEq/l)	4.3 (3.9–4.8)
BUN (mg/dl)	36.4 (20.875–61.5)
Creatinine (mg/dl)	1.51 (0.98–2.2825)
Lactate (mmol/l)	2.85 (1.975–4.825)
HCO_3_^-^ (mmol/l)	19.95 (15.925–23.5)
CRP (mg/dl)	9.995 (1.6175–25.3175)
Blood glucose level (mg/dl)	158.5 (104–229.75)
AT3 (%)	79.5 (64–88.5)
UA (mg/dl)	7.4 (5.5–9.95)

(B)		
**Site of Infection**	**n = 58**	**Pathogen**
Pulmonary, n (%)	31 (53.4%)	
Pure gram negative, n (%)	7 (12.1%)	*Escherichia coli*, *Haemophilus influenzae*, *Klebsiella pneumoniae*, *Pseudomonas aeruginosa*
Pure gram positive, n (%)	8 (13.8%)	*Staphylococcus aureus*, *Streptococcus agalactiae*
Mixed bacteria, n (%)	9 (15.5%)	*Klebsiella pneumoniae*, *Escherichia coli*, *Streptococcus pneumoniae*, *Streptococcus anginosus*, *Enterobacter cloacae*
Influenza virus, n (%)	2 (3.4%)	
*Candida albicans*, n (%)	1 (1.7%)	
No pathogen, n (%)	4 (6.9%)	
Positive blood culture, n (%)	3 (5.2%)	
Urinary tract, n (%)	15 (25.9%)	
Pure gram negative, n (%)	10 (17.3%)	*Escherichia coli*, *Klebsiella pneumoniae*, *Citrobacter koseri*
Pure gram positive, n (%)	3 (5.2%)	*Staphylococcus aureus*, *Staphylococcus epidermidis*, *Enterococcus raffinosus*
Mixed bacteria, n (%)	1 (1.7%)	*Escherichia coli*, *Staphylococcus epidermidis*
No pathogen, n (%)	1 (1.7%)	
Positive blood culture, n (%)	7 (12.1%)	
Intra-abdominal, n (%)	6 (10.3%)	
Pure gram negative, n (%)	3 (5.2%)	*Escherichia coli*, *Pseudomonas aeruginosa*
Pure gram positive, n (%)	1 (1.7%)	*Streptococcus gordonii*
Mixed bacteria, n (%)	2 (3.4%)	*Klebsiella pneumoniae*, *Streptococcus faecalis*, *Citrobacter koseri*
Positive blood culture, n (%)	3 (5.2%)	
Soft tissue, n (%)	3 (5.2%)	
Pure gram positive, n (%)	3 (5.2%)	*Streptococcus agalactiae*, *Staphylococcus aureus*, *Staphylococcus epidermidis*
Positive blood culture, n (%)	2 (3.4%)	
Others, n (%)	3 (5.2%)	
Pure gram positive, n (%)	2 (3.4%)	*Staphylococcus aureus*
Mixed bacteria, n (%)	1 (1.7%)	*Streptococcus faecalis*, *Streptococcus constellatus*
Positive blood culture, n (%)	2 (3.4%)	

Abbreviations: *ALT* alanine aminotransferase, *APACHE* Acute Physiology and Chronic Health Evaluation, *AST* aspartate aminotransferase, *AT* antithrombin, *BUN* blood urea nitrogen, *CRP* C-reactive protein, *DIC* disseminated intravascular coagulation, *SOFA* Sequential Organ Failure Assessment, *UA* uric acid, *WBC* white blood cell.

Nonparametric data are presented as median with interquartile range. Categorical data are presented as n (%). The biochemical data in this table are based on data at the time of admission (i.e., at the time of sepsis diagnosis). The “other” category included one case each of periapical dental infection, infectious endocarditis, and bacteremia with unknown foci.

### Brain volume reduction

**Fig 2:**
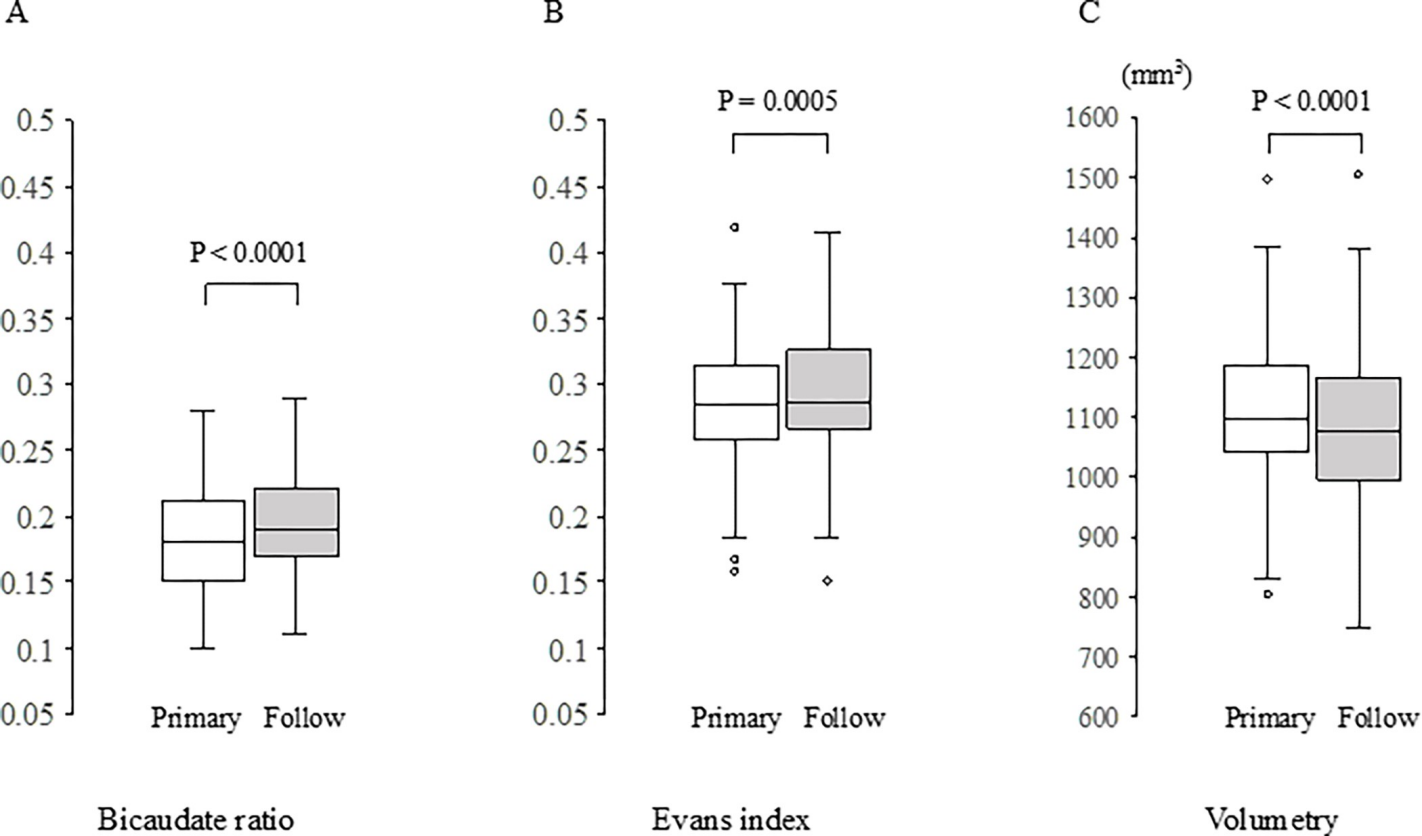
Comparison of brain volume reduction assessed using head computed tomography between admission and follow-up. (A) Change in bicaudate ratio. (B) Change in the Evans index. (C) Change in brain volume by volumetry. The Wilcoxon signed-rank test was performed to compare the two groups of paired nonparametric data. *P < 0.05.

The mean timepoint at which the second head CT was performed was the 13^th^ (interquartile range, 7–17) day of hospitalization. A comparison between the findings from the two timepoints of head CT showed a significant increase in BCR in 38/58 (65.5%, *P* < 0.0001) patients, significant increase in EI in 35/58 (60.3%, *P* = 0.0005), and significant decrease in Vo in 46/58 (79.3%, *P* < 0.0001) (Fig 2). Regarding the extent of brain volume changes, BCR increased by 5.9% (interquartile range, 0–15.7), EI increased by 1.4% (interquartile range, 0–3.6), and Vo decreased by 1.8% (interquartile range, 0.3–4.5) compared with the values on admission.

### Association between brain volume and type of bacteria, bacteremia, APACHE II score, SOFA score, and shock

There was no difference in the degree of decrease in brain volume depending on the type of initiating organism or the presence or absence of bacteremia. The Steel–Dwass test for BCR, EI, and Vo change rates based on infection foci showed no differences in brain volume changes. No significant differences were found between the groups with and without bacteremia in terms of the percentage changes in BCR, EI, and Vo (BCR: *P* = 0.2235, EI: *P* = 0.2883, Vo: *P* = 0.1141). There were significant differences in APACHE II scores (*P* = 0.043), SOFA scores at admission (*P* = 0.0387), and the presence or absence of shock (*P* = 0.012) between groups with and without decreased brain volume for BCR, but not for EI and Vo (Table 2). There were no significant differences in the worst SOFA scores between groups with and without decreased brain volume (Table 2).

**Table 2:** Differences in background and neurological outcomes between patients with and without brain volume reduction.

	Bicaudate ratio			Evans index			Volumetry		
	Brain Volume Reduction (n = 38)	No Brain Volume Reduction (n = 20)	*P*-value	Brain Volume Reduction (n = 35)	No Brain Volume Reduction (n = 23)	*P*-value	Brain Volume Reduction (n = 46)	No Brain Volume Reduction (n = 12)	*P*-value
Age (years)	78 (68–86)	75.5 (70–82.5)	0.9022	78 (69–86)	76 (68–84)	0.5088	76 (70–86)	76.5 (64.5–82.25)	0.403
APACHE Ⅱ score at admission	23 (19.5–27)	27 (21.5–35.75)	0.043	24 (21–27)	24 (17–35)	0.65	24 (21–29)	24.5 (19.5–31)	0.9005
SOFA score at admission	6.5 (5–9.25)	8 (6.25–10)	0.0387	7 (5–10)	7 (6–10)	0.7008	7 (5–10)	8 (6–11.5)	0.4224
Worst SOFA score (Average hospitalization day 2)	7 (4.75–10)	8 (6.25–11.5)	0.1127	7 (5–10)	8 (6–10)	0.3220	7 (5–10)	7 (5.25–11.5)	0.9615
Septic shock, n (%)	7 (18.4)	10 (50)	0.012	9 (25.7)	8 (34.8)	0.458	12 (26.1)	5 (41.7)	0.291
Days in the intensive care unit	8 (4.75–13.25)	9 (7.25–12)	0.2064	10 (4–14)	8 (6–10)	0.6211	10 (6–17)	8 (4.25–11.25)	0.4077
Duration of intubation (days)	3 (0–6)	0.5 (0–5.75)	0.6678	3 (0–9)	2 (0–5)	0.611	3 (0–12)	0 (0–5.75)	0.2192
DIC at admission, n (%)	7 (18.4)	7 (35)	0.1608	9 (25.7)	5 (21.7)	0.7293	10 (21.7)	4 (33.3)	0.4032
Katz index at discharge	2.5 (0–6)	1.0 (0–2.75)	0.0623	1.0 (0–4)	3.0 (0–5)	0.2261	1.5 (0–5)	1.0 (0–2.5)	0.3816
WBC (×10^3^/μl)	10.55 (7.25–15.6)	11.5 (8.125–17.4)	0.7373	10.6 (6.6–15.5)	11.2 (8.1–16.2)	0.4942	10 (7.1–13.1)	14.4 (5.55–16.925)	0.977
Hemoglobin (g/dl)	13.2 (11.475–14.5)	13.05 (11–15.4)	0.8315	12.9 (11.4–14.5)	13.3 (11.4–15.3)	0.5888	12.8 (11.4–14.4)	15.05 (11.925–15.825)	0.0536
Hematocrit (%)	40.05 (34.475–43.4)	40.95 (33.1–45.75)	0.5449	39.8 (33.4–43.4)	40.9 (34.4–44.2)	0.4892	39.9 (34.5–43.4)	43.5 (38.4–45.85)	0.0381
Platelet (×10^4^/μl)	18.2 (12.0–27.9)	18.8 (10.7–29.475)	0.8829	18.1 (12–27.9)	20.3 (11–30.1)	0.413	21.3 (14.1–28.4)	19.7 (10.575–31.75)	0.6868
Albumin (g/dl)	3.3 (2.7–3.9)	3.25 (2.725–3.675)	0.5663	3.2 (2.7–3.9)	3.4 (2.9–3.7)	0.65	3.4 (2.7–3.9)	3.15 (2.55–3.775)	0.5384
T.Bilirubin (mg/dl)	0.65 (0.4575–0.985)	0.675 (0.4–1.53)	0.9023	0.76 (0.49–1.1)	0.53 (0.38–0.99)	0.2831	0.65 (0.36–0.94)	0.93 (0.47–2.16)	0.2376
AST (U/l)	42 (24–58.25)	77 (29.5–378.75)	0.5663	51 (26–93)	34 (22–83)	0.6221	30 (24–53)	82 (48–378.75)	0.0502
ALT (U/l)	24 (13–47)	34 (18–273)	0.1301	30 (13–74)	27 (15–50)	0.8549	19 (13–35)	77 (28.5–176.25)	0.0307
Na (mEq/l)	137 (133.75–141.25)	141.5 (136–146)	0.0405	138 (134–141)	140 (136–146)	0.0603	140 (136–144)	139 (136.25–145.5)	0.4305
K (mEq/l)	4.3 (3.875–4.8)	4.4 (3.9–5.275)	0.4709	4.5 (3.9–4.8)	4.3 (3.8–4.7)	0.4936	4.5 (3.9–4.8)	4.5 (4.025–5.2)	0.403
BUN (mg/dl)	28.25 (18.525–47.325)	63.7 (31.375–101.25)	0.0014	31.5 (20.5–52.3)	46.9 (22.5–69)	0.2459	35.5 (22.5–58.5)	47.7 (23.2–101.25)	0.1669
Creatinine (mg/dl)	1.43 (0.91–2.0825)	1.895 (1.29–3.8875)	0.0622	1.42 (0.98–2.15)	1.7 (0.98–4.19)	0.2941	1.47 (0.91–2.27)	1.8 (1.2775–4.14)	0.1177
Lactate (mmol/l)	2.75 (1.975–4.075)	2.95 (1.9–6.8)	0.6234	3.1 (2.1–4.9)	2.6 (1.4–4)	0.332	2.8 (2.2–4.8)	5.05 (2.3–7.125)	0.0757
HCO_3_^-^ (mmol/l)	20.5 (16.175–23.625)	18.5 (13.825–23.225)	0.2624	20.3 (16.5–23.5)	19.6 (11.1–23.5)	0.8301	20.4 (16.1–23.1)	18.5 (14.475–22.15)	0.2823
CRP (mg/dl)	6.55 (0.635–22.2675)	16.9 (3.79–27.205)	0.0936	13.39 (1.09–25.29)	9.99 (1.65–26.09)	0.8239	3.12 (0.47–18.93)	17.78 (6.3–28.86)	0.0758
Blood glucose level (mg/dl)	164 (108.75–220.75)	147 (93.5–348.75)	0.8829	157 (98–220)	168 (108–289)	0.2833	156 (109–289)	166.5 (98.5–189.75)	0.6798
AT3 (%)	77.5 (64–87.25)	80.5 (62.5–93.75)	0.8894	72 (60–88)	83 (69–95)	0.169	84 (68–95)	80.5 (65.5–89.25)	0.4255
UA (mg/dl)	7.2 (5.5–9.5)	7.95 (5.25–16.05)	0.2875	7.45 (5.425–8.975)	7.2 (5.5–16.4)	0.3994	7.4 (6.1–9.5)	7.45 (5.05–18.875)	0.5711
Fluid balance for 24 after admission	2278 (1217.5–3420.5)	2687.5(1325.25–5013.25)	0.254	2326 (1162–3775)	2258 (1380–3817.25)	0.8217	2238 (1311–3521)	2825 (1146.25–6404)	0.2702
Fluid balance on the day of the final CT	444(150–623.5)	398.5 (11.5–561.5)	0.3644	430 (118–607)	459.5 (133.75–636)	0.7537	444 (140–593)	419 (92.5–624.75)	0.9614

Abbreviations: *ALT* alanine aminotransferase, *APACHE* Acute Physiology and Chronic Health Evaluation, *AST* aspartate aminotransferase, *AT* antithrombin, *BUN* blood urea nitrogen, *CRP* C-reactive protein, *CT* computed tomography, *DIC* disseminated intravascular coagulation, *SOFA* Sequential Organ Failure Assessment, *UA* uric acid, *WBC* white blood cell.

Nonparametric data are presented as median with interquartile range. Categorical data are presented as n (%). *P*-value <0.05 is considered statistically significant.

### Association between brain volume reduction and ADL

**Fig 3:**
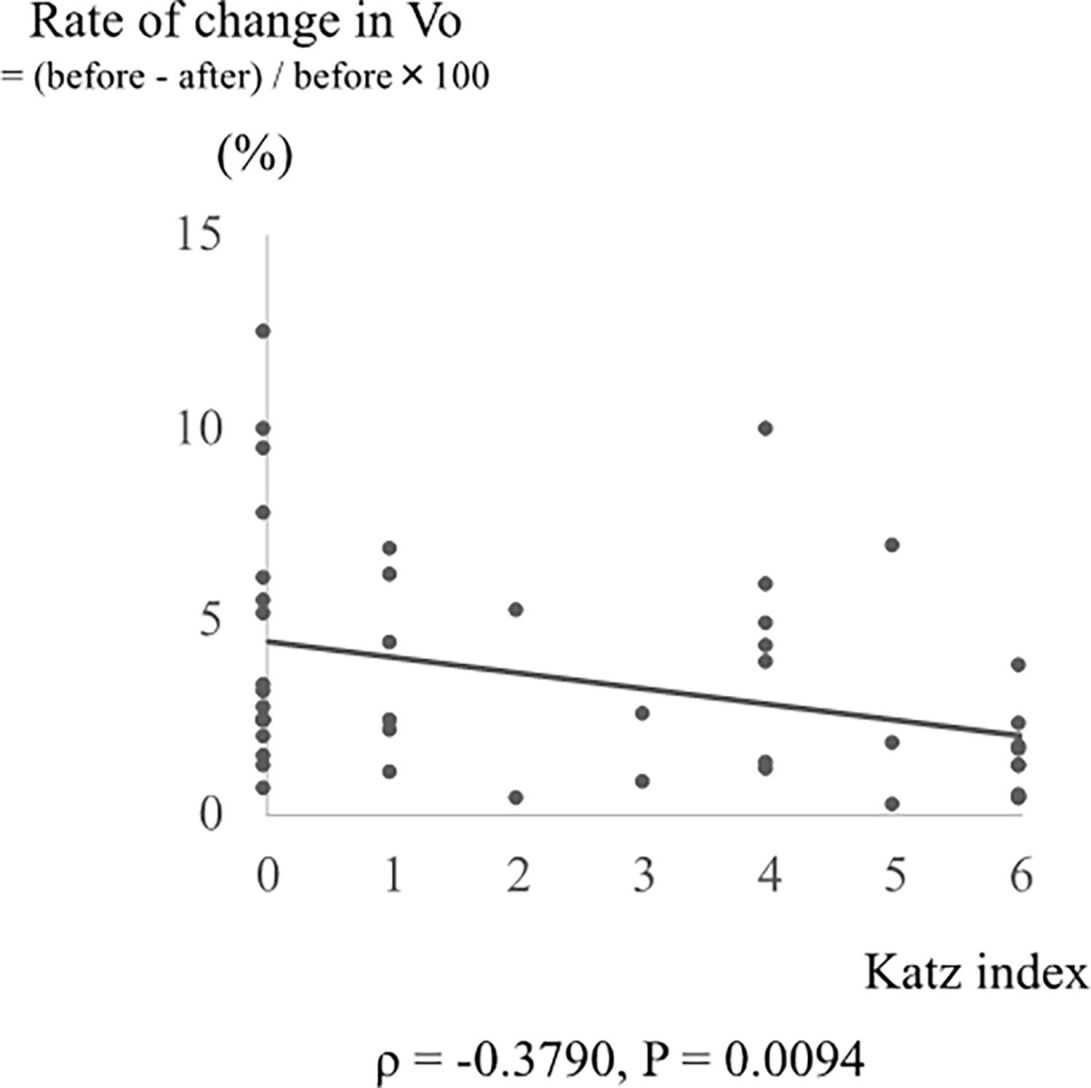
Association between the degree of brain volume reduction and activities of daily living function. Correlation between rate of change in the brain volume by volumetry (Vo) and the Katz index. Spearman’s rank correlation test was performed to analyze the correlation between the rate of change in Vo and the Katz index. The coefficient of correlation is shown by ρ, *P < 0.05. The reference line shows a positive correlation or tendency.

The examination of 58 patients, including those without brain volume reduction, revealed no significant correlation between the rates of change in BCR/EI/Vo and the Katz index. However, when the examination was limited to patients with decreased brain volume, a significant correlation was found between the rates of change in BCR/EI/Vo and the Katz index. The correlation between the rates of changes in the BCR, EI, Vo and Katz index was examined in 38 patients with increased BCR, 35 patients with increased EI, and 46 patients with decreased Vo. A significant correlation was found between the rate of change in Vo and the Katz index (ρ = −0.3790, *P* = 0.0094) (Fig 3).

The comparison of demographic characteristics between the two groups—one with and the other without brain volume reduction—showed that the APACHE II score (*P* = 0.0430), the SOFA score (*P* = 0.0387), septic shock (*P* = 0.0120), serum sodium level (*P* = 0.0405), and serum urea nitrogen level (*P* = 0.0014) differed according to the BCR, whereas the serum hematocrit value (*P* = 0.0381) and ALT level (*P* = 0.0307) differed according to Vo (Table 2). Regarding volumetry, the median Katz index for the brain volume reduction group was 1.5 and that for the group with no brain volume reduction was 1.0 (Table 2). However, there was no significant difference between the two groups (*P* = 0.3816). Furthermore, the mean (± standard deviation) of the Katz index was 2.46 ± 2.40 for the brain volume reduction group and 1.58 ± 2.14 for the group without brain volume reduction, indicating high variability.

### Assessment of fluid balance

The in-out fluid balance for 24 h after admission and on the day of the final CT was compared between the two groups with and without brain volume reduction. There were no significant differences between the two groups in BCR, EI, and Vo (Table 2).

## Discussion

This prospective study had several key findings. Based on the head CT findings on admission, we found that brain volume was reduced during the course of sepsis. In addition, in patients with progressive brain volume reduction, there was a significant positive correlation between the rate of brain volume changes and the Katz index, which measures ADL function. Previous reports have shown that some patients have decreased brain volume during the acute phase in sepsis [13,14]. A brain MRI study in patients with sepsis and neurological changes after admission to the ICU showed that approximately 16% of patients had brain volume reduction [14]. In addition, some MRI abnormalities (acute cerebral infarction 22.6%, white matter lesions 16.1%) were observed in half of the patients [13,23,24]. In studies that examined the volume of each part of the brain in patients with cerebral dysfunction associated with sepsis, the most significant reduction in brain volume was found in the white matter of the cerebrum; however, the deep gray matter and cerebellar cortex were relatively unaffected [25–27]. As the present results differed depending on the method used to measure brain volume reduction, it was not possible to examine which part of the brain was reduced in volume. The reason for this could be that BCR and EI evaluate the width between the anterior horns in a flat plane, while Vo evaluates the entire brain in a three-dimensional plane. Moreover, in a study of patients with sepsis-associated encephalopathy, a significant decrease in brain volume was observed in coma patients [28]. However, none of those previous reports studied the time course starting from the acute phase [13,14], and most were comparisons with healthy volunteers [24,26,29,30]. Although brain MRI is the best method for evaluating the brain during sepsis [31], given the extreme difficulty of performing frequent MRI during the systemic management of sepsis, we used head CT in this study.

We also assessed whether fluid balance affected the reduction in brain volume. There were no significant differences in fluid balance for 24 h after admission and on the day of the final CT between the groups with and without brain volume reduction. Specifically, brain volume reduction had no causal relationship with fluid balance or cerebral edema.

Brain volume reduction in the early phase in patients with sepsis could indicate a clinical predisposition for developing non-favorable ADL function, with increased risk of post-intensive care syndrome. The Katz index was used to evaluate ADL performance in this study. This index is widely used in clinical studies to assess hospital-associated disability, meaning a decrease in ADL function associated with hospitalization [22]. However, we did not evaluate long-term ADL function; further studies are needed to identify whether brain volume reduction could be a novel target for sepsis-associated encephalopathy. A multicenter study reported that approximately half of the patients with sepsis had sepsis-associated encephalopathy [32]; thus, sepsis-associated encephalopathy might be related to the mechanism of brain volume reduction.

Brain volume reduction has been reported to occur between the 9^th^ and 12^th^ days of onset in patients with pediatric acute encephalopathy [33] and to begin on the 3^rd^ day after head trauma [34]. Even in the acute phase of ICU treatment, various factors such as sepsis may contribute to brain volume reduction. Brain atrophy is an irreversible change [35], but the design of this study is insufficient to conclude whether the volume reduction observed corresponded to brain atrophy, as this variable was not examined over the long term. Especially in sepsis, it is well known that consciousness disorder is complicated, and it is considered to be a diffuse brain disorder resulting from systemic inflammatory response syndrome secondary to infection [36], although the exact mechanism has been unclear. In recent years, such a condition has been called sepsis-associated encephalopathy and is regarded as an important pathological condition associated not only to survival rates but also to the rate of reintegration into society [6,7]. Therefore, to improve the outcome of patients with sepsis, it is necessary not only to reduce the mortality rate but also to improve this rate of reintegration, including long-term neurological outcomes such as ADL function. Importantly, the present study revealed that patients with sepsis and brain volume reduction from the initial stage of ICU admission had decreased ADL function relative to neurological outcomes at discharge or transfer. By clarifying the mechanism of brain volume reduction in sepsis, we believe that a new treatment strategy for improving the rate of reintegration into society may be developed in the future.

The present study investigated possible reasons for brain volume reduction during the acute phase of sepsis, assuming an influence of the severity of sepsis, bacterial species, and cerebral ischemia caused by hypotension or hypoxia during management (secondary brain damage due to cerebral ischemia). Regarding the severity of illness, a statistical analysis of the relationship between SOFA scores (as a metric for the severity of sepsis at admission and at worst during the disease course) and hypoxia or hypotension (because the SOFA score includes items for hypotension and hypoxia) yielded a significant relationship between SOFA scores at admission and brain volume reduction for BCR, not the other index. Moreover, we examined whether the type of bacteria and the presence of bacteremia were related to decreases in brain volume. Orhun et al. [14] reported that significantly more patients with sepsis-induced brain dysfunction with brain lesions had positive blood culture results; however, no significant differences in brain atrophy were observed in this study. Further, various effects could also be ruled out, such as direct effects on brain cells by systemic inflammatory mediators that occur during sepsis [37] and volume reduction due to hyperglycemia, inflammatory cell infiltration, and cell phagocytosis in ischemic brain tissue [38]. However, these effects have not been clarified in the present study. A more detailed analysis of ICU data is needed in the future, including the measurement of inflammatory mediators (such as cytokines), the duration of hypotension and hypoxia, and sustained hyperglycemia.

There are some limitations to this study. The first is the absence of a control group. It is unclear whether the results were associated with sepsis-specific decreased brain volume or other medical conditions (such as hypotension and hypoxia). It would have been beneficial to include a control group without sepsis. Second, the median age of patients in this study was 79 years and, therefore, the included patients were elderly. The specific characteristics of sepsis in older patients may have influenced the results. Therefore, whether the results can be generalized to younger individuals warrants further investigation. Third, the number of patients was limited in this single-center study, and the most severe patients, who died early (n = 19), were not examined. In addition, repeated head CT is burdensome for patients, and a large number of patients were unable to provide consent, making the sample size of this study small. Fourth, the evaluation period was short. The median evaluation time for brain volume was the 13th day of hospitalization, and the median evaluation time for ADL was the 20th day of hospitalization. Further studies are needed to determine if the acute effects are consistent with long-term ADL function after intensive care. Fifth, the changes in brain volume reduction in our study were small, and the implications of these changes may have been underexamined, such as by not examining long-term ADL performance. Specifically, it has not been possible to examine whether this volume reduction was organic and related to long-term outcomes. In the future, it is necessary to examine the exact location and persistence of the brain volume reduction in the long term. Sixth, it is difficult to identify the time of onset of sepsis [39]. Therefore, as our reference date was the time of admission, there is a possibility that the evaluation date of head CT may vary. Finally, the assessment of brain volume reduction was assessed from head CT instead of brain MRI. In the acute phase of critically ill patients, their condition is often unstable, and it is difficult to safely perform MRI at the time of admission and follow-up. Therefore, we used head CT instead, for the reasons previously explained.

## Conclusion

Brain volume reduction occurred in the acute phase of sepsis in 55%–79% of elderly patients. Brain volume reduction in the acute phase of sepsis was associated with poor ADL function, although the clinical significance of this phenomenon remains unclear. Future studies aimed at clarifying the underlying causes and possible countermeasures will help improve not only survival but also long-term ADL function in patients with sepsis.

## Supporting information

S1 Data (XLSX)

## References

[1] Vincent JL, Marshall JC, Ñamendys-Silva SA, François B, Martin-Loeches I, Lipman J, et al. Assessment of the worldwide burden of critical illness: the Intensive Care Over Nations (ICON) audit. Lancet Respir Med. 2014;2: 380–386. doi: 10.1016/S2213-2600(14)70061-X 24740011

[2] Needham DM, Bronskill SE, Calinawan JR, Sibbald WJ, Pronovost PJ, Laupacis A. Projected incidence of mechanical ventilation in Ontario to 2026: preparing for the aging baby boomers. Crit Care Med. 2005;33: 574–579. doi: 10.1097/01.ccm.0000155992.21174.31 15753749

[3] Iwashyna TJ, Cooke CR, Wunsch H, Kahn JM. Population burden of long-term survivorship after severe sepsis in older Americans. J Am Geriatr Soc. 2012;60: 1070–1077. doi: 10.1111/j.1532-5415.2012.03989.x 22642542 PMC3374893

[4] Oeyen SG, Vandijck DM, Benoit DD, Annemans L, Decruyenaere JM. Quality of life after intensive care: a systematic review of the literature. Crit Care Med. 2010;38: 2386–2400. doi: 10.1097/CCM.0b013e3181f3dec5 20838335

[5] Mayr FB, Yende S, and Angus DC. Epidemiology of severe sepsis. Virulence. 2014;5: 4–11. doi: 10.4161/viru.27372 24335434 PMC3916382

[6] Iacobone E, Bailly-Salin J, Polito A, Friedman D, Stevens RD, Sharshar T. Sepsis-associated encephalopathy and its differential diagnosis. Crit Care Med. 2009;37 Suppl: S331–S336. doi: 10.1097/CCM.0b013e3181b6ed58 20046118

[7] Mazeraud A, Pascal Q, Verdonk F, Heming N, Chrétien F, Sharshar T. Neuroanatomy and physiology of brain dysfunction in sepsis. Clin Chest Med. 2016;37: 333–345. doi: 10.1016/j.ccm.2016.01.013 27229649

[8] Needham DM, Davidson J, Cohen H, Hopkins RO, Weinert C, Wunsch H, et al. Improving long-term outcomes after discharge from intensive care unit: report from a stakeholders’ conference. Crit Care Med. 2012;40: 502–509. doi: 10.1097/CCM.0b013e318232da75 21946660

[9] Schott JM, Price SL, Frost C, Whitwell JL, Rossor MN, Fox NC. Measuring atrophy in Alzheimer disease: a serial MRI study over 6 and 12 months. Neurology. 2005;65: 119–124. doi: 10.1212/01.wnl.0000167542.89697.0f 16009896

[10] Nestor SM, Rupsingh R, Borrie M, Smith M, Accomazzi V, Wells JL, et al. Ventricular enlargement as a possible measure of Alzheimer’s disease progression validated using the Alzheimer’s Disease Neuroimaging Initiative database. Brain. 2008;131: 2443–2454. doi: 10.1093/brain/awn146 18669512 PMC2724905

[11] van Zagten M, Kessels F, Boiten J, Lodder J. Interobserver agreement in the assessment of cerebral atrophy on CT using bicaudate and sylvian-fissure ratios. Neuroradiology. 1999;41: 261–264. doi: 10.1007/s002340050743 10344510

[12] Chrzan R, Gleń A, Bryll A, Urbanik A. Computed tomography assessment of brain atrophy in centenarians. Int J Environ Res Public Health. 2019;16: 3659–3669. doi: 10.3390/ijerph16193659 31569457 PMC6801833

[13] Suchyta MR, Jephson A, Hopkins RO. Neurologic changes during critical illness: brain imaging findings and neurobehavioral outcomes. Brain Imaging Behav. 2010; 4:22–34. doi: 10.1007/s11682-009-9082-3 20503111

[14] Orhun G, Esen F, Özcan PE, Sencer S, Bilgiç B, Ulusoy C, et al. Neuroimaging findings in sepsis-induced brain dysfunction: association with clinical and laboratory findings. Neurocrit Care, 2019;30: 106–117. doi: 10.1007/s12028-018-0581-1 30027347

[15] Rhodes A, Evans LE, Alhazzani W, Levy MM, Antonelli M, Ferrer R, et al. Surviving Sepsis Campaign: international guidelines for management of sepsis and septic shock: 2016. Intensive Care Med. 2017;43: 304–377. doi: 10.1007/s00134-017-4683-6 28101605

[16] Katz S, Ford AB, Moskowitz RW, Jackson BA, Jaffe MW. Studies of illness in the aged. The Index of ADL: A standardized measure of biological and psychosocial function. JAMA. 1963;185: 914–919. doi: 10.1001/jama.1963.03060120024016 14044222

[17] Gomori JM, Steiner I, Melamed E, Cooper G. The assessment of changes in brain volume using combined linear measurements: a CT-scan study. Neuroradiology. 1984;26: 21–24. 6738838 10.1007/BF00328197

[18] Evans WA. An encephalographic ratio for estimating ventricular enlargement and cerebral atrophy. Arch NeurPsych. 1942;47: 931–937.

[19] Annen J, Frasso G, Crone JS, Heine L, Di Perri C, Martial C, et al. Regional brain volumetry and brain function in severely brain-injured patients. Ann Neurol. 2018;83: 842–853. doi: 10.1002/ana.25214 29572926

[20] Yamamoto Y, Mihara M, Yoshizawa T, Sugenoya S, Makino A, Gomi K, et al. Analysis of portal vein embolization using absolute ethanol before major hepatectomy. Int Surg. 2016;101: 453–457.

[21] Katz S, Downs TD, Cash HR, Grotz RC. Progress in development of the index of ADL. Gerontologist. 1970;10: 20–30. doi: 10.1093/geront/10.1_part_1.20 5420677

[22] Loyd C, Markland AD, Zhang Y, Fowler M, Harper S, Wright NC, et al. Prevalence of hospital-associated disability in older adults: a meta-analysis. J Am Med Dir Assoc. 2020;21: 455–461.e5. doi: 10.1016/j.jamda.2019.09.015 31734122 PMC7469431

[23] Polito A, Eischwald F, Maho AL, Polito A, Azabou E, Annane D, et al. Pattern of brain injury in the acute setting of human septic shock. Crit Care. 2013;17: R204. doi: 10.1186/cc12899 24047502 PMC4057119

[24] Gunther ML, Morandi A, Krauskopf E, Pandharipande P, Girard TD, Jackson JC, et al. The association between brain volumes, delirium duration, and cognitive outcomes in intensive care unit survivors: the VISIONS cohort magnetic resonance imaging study. Crit Care Med. 2012;40: 2022–2032. doi: 10.1097/CCM.0b013e318250acc0 22710202 PMC3697780

[25] Gofton TE, Young GB. Sepsis-associated encephalopathy. Nat Rev Neurol. 2012;8: 557–566. doi: 10.1038/nrneurol.2012.183 22986430

[26] Adam N, Kandelman S, Mantz J, Chrétien F, Sharshar T. Sepsis-induced brain dysfunction. Expert Rev Anti Infect Ther. 2013;11: 211–221. doi: 10.1586/eri.12.159 23409826

[27] Mazeraud A, Righy C, Bouchereau E, Benghanem S, Bozza FA, Sharshar T. Septic-associated encephalopathy: a comprehensive review. Neurotherapeutics. 2020;17: 392–403. doi: 10.1007/s13311-020-00862-1 32378026 PMC7283452

[28] Orhun G, Tüzün E, Bilgiç B, Ergin Özcan P, Sencer S, Barburoğlu M, et al. Brain volume changes in patients with acute brain dysfunction due to sepsis. Neurocrit Care. 2020;32: 459–468. doi: 10.1007/s12028-019-00759-8 31187433

[29] Sharshar T, Carlier R, Bernard F, Guidoux C, Brouland JP, Nardi O, et al. Brain lesions in septic shock: a magnetic resonance imaging study. Intensive Care Med. 2007;33: 798–806. doi: 10.1007/s00134-007-0598-y 17377766

[30] Semmler A, Widmann CN, Okulla T, Urbach H, Kaiser M, Widman G, et al. Persistent cognitive impairment, hippocampal atrophy and EEG changes in sepsis survivors. J Neurol Neurosurg Psychiatry. 2013;84: 62–69. doi: 10.1136/jnnp-2012-302883 23134661

[31] Oddo M and Taccone FS. How to monitor the brain in septic patients? Minerva Anestesiol. 2015;81: 776–788. 25812488

[32] Sonneville R, de Montmollin E, Poujade J, Garrouste-Orgeas M, Souweine B, Darmon M, et al. Potentially modifiable factors contributing to sepsis-associated encephalopathy. Intensive Care Med. 2017;43: 1075–1084. doi: 10.1007/s00134-017-4807-z 28466149

[33] Lo TYM, McPhillips M, Minns RA, Gibson RJ. Cerebral atrophy following shaken impact syndrome and other non-accidental head injury (NAHI). Pediatr Rehabil. 2003;6: 47–55. doi: 10.1080/1363849031000109516 12745895

[34] Greer JE, McGinn MJ, Povlishock JT. Diffuse traumatic axonal injury in the mouse induces atrophy, c-Jun activation, and axonal outgrowth in the axotomized neuronal population. J Neurosci. 2011;31: 5089–5105. doi: 10.1523/JNEUROSCI.5103-10.2011 21451046 PMC3076099

[35] Marion CM, McDaniel DP, Armstrong RC. Sarm1 deletion reduces axon damage, demyelination, and white matter atrophy after experimental traumatic brain injury. Exp Neurol. 2019;321: 113040. doi: 10.1016/j.expneurol.2019.113040 31445042

[36] Eidelman LA, Putterman D, Putterman C, Sprung CL. The spectrum of septic encephalopathy. Definitions, etiologies, and mortalities. JAMA. 1996;275: 470–473. 8627969

[37] Deutschman CS, Tracey KJ. Sepsis: current dogma and new perspectives. Immunity. 2014;40: 463–475. doi: 10.1016/j.immuni.2014.04.001 24745331

[38] Lin B, Ginsberg MD, Busto R, Li L. Hyperglycemia triggers massive neutrophil deposition in brain following transient ischemia in rats. Neurosci Lett. 2000;278: 1–4. doi: 10.1016/s0304-3940(99)00889-7 10643786

[39] Abe T, Kushimoto S, Tokuda Y, Phillips GS, Rhodes A, Sugiyama T, et al. Implementation of earlier antibiotic administration in patients with severe sepsis and septic shock in Japan: a descriptive analysis of a prospective observational study. Crit Care. 2019;23: 360. doi: 10.1186/s13054-019-2644-x 31744549 PMC6862854

