# Supplementary material for: Relationship between brain volume reduction during the acute phase of sepsis and activities of daily living in elderly patients: A prospective cohort study
Source: PLoS One. 2023 May 16;18(5):e0284886. doi: 10.1371/journal.pone.0284886 (PMC10187932; doi:10.1371/journal.pone.0284886)
Supplement: Attachment — Submitted filename: Review_Plos one.docx [file pone.0284886.s002.docx]

Toru Hosokawa *et al.* showed the association between brain volume reduction and performance of the activities of daily living via the Katz index. Overall, this is a concise study with a limited number of participants with sepsis or septic shock. There are some points that the authors need to revise.

(Major)

P5 Ln 83 Blood test for what? In Line 83, authors described that blood tests were performed several times, but in Line132, only the results at the time of admission were used for examination.

So, are the biochemical test results used throughout the result (Table 1) at the time of admission?

Wouldn't it be more desirable to process the statistics in Table 2 using the biochemical results at the time of sepsis diagnosis or CT scan performed? It is better to describe when the result of the biochemical test (Table 2) was obtained.

P6 Ln91-93 What does it mean that “samples from the suspected site of infection, diagnostic imaging, and examination data were collected, ----- and bacterial culture results”? Please clarify the statement.

P14 Table1(B) Clarify the "others” (n = 3)

P14 Table1(C) It would be better to add more combined information for site of infection and bacteria identification results. In addition, if there was “no pathogen”, but is suspected of infection, additional description is required, and the reason for why no pathogen is also required(e.g., Improper specimen, use of antibiotics prior to specimen collection). Since it seems meaningless to present the number of simple “pure gram negative” and “pure gram positive” as Table 1(c), it is better to describe a table that show which bacteria were identified in which part by combining 1(B) and 1(C).

P17 Ln231-236 There is no description in the discussion about why some demographic characteristics show differences according to BCR and VO. The possible causes for this difference should be described in the Discussion section.

Was there any association with brain volume reduction in terms of bacterial isolation from any infection site? Or was there no relationship between bacteremia and brain volume reduction?

(Minor)

Change "P" in P-values to italic.

Typographical error in Table 2 (p value for platelet in Bicaudate ratio
